# Does the Valuation of Nutritional Claims Differ among Consumers? Insights from Spain

**DOI:** 10.3390/nu9020132

**Published:** 2017-02-13

**Authors:** Francesc Jurado, Azucena Gracia

**Affiliations:** 1 Unidad de Economía Agroalimentaria, Centro de Investigación y Tecnología Agroalimentaria de Aragón (CITA), Gobierno de Aragón, Avda. Montañana, 930, 50059 Zaragoza, Spain; fjurado@aragon.es; 2 Instituto Agroalimentario de Aragón—IA2 (CITA-Universidad de Zaragoza), 50059 Zaragoza, Spain

**Keywords:** choice experiment, heterogeneous preferences, nutritional claims

## Abstract

The presence in the market of food products with nutritional claims is increasing. The objective of this paper is to assess consumers’ valuation of some nutritional claims (‘high in fiber’ and ‘reduced saturated fat’) in a European country and to test for differences among consumers. An artefactual non-hypothetical experiment was carried out in a realistic setting (mock/real brick-and-mortar supermarket) with a sample of 121 Spanish consumers stratified by gender, age, and body mass index. A latent class model was specified and estimated with the data from the experiment. Results indicate that consumers positively valued both nutritional claims, but the valuation was heterogeneous, and three consumer segments were detected. Two of them positively valued both nutritional claims (named ‘nutritional claim seekers’), while the third segment’s valuation was negative (named ‘nutritional claim avoiders’). This last segment is characterized by being younger males with university studies who give the least importance to health, natural ingredients, and the calorie/sugar/fat content when shopping. They pay less attention to nutritional information, and they stated that they use this information to a lesser extent. These consumers showed the least interest in healthy eating, and they reported that they do not have health problems related to their diet.

## 1. Introduction

Within the European context, chronic diseases constitute the main cause of death, the most important ones being heart disease, cerebrovascular disease, cancer, and diabetes. The most significant preventable risk factors are unhealthy diet, physical inactivity, and tobacco use [1]. Moreover, it is widely accepted that current eating habits in general tend to be less than healthy. One reason for this phenomenon is the increase in the consumption of pre-prepared or quick-to-prepare foods and a subsequent reduction in consumers who cook food every day from fresh raw ingredients [2]. As a consequence, in many countries, dietary recommendations and actual food intake do not coincide at the general population level [3,4,5]. Thus, a change in dietary behavior is crucial to reduce the effect of unhealthy diets on consumers’ health. One way to improve the quality of consumers’ diets is to increase the consumption of fiber and decrease the consumption of fat, in particular saturated fat, to reduce the risk of chronic disease [4,6] by eating more fresh food products (fruits and vegetables), and cooking food at home from healthier ingredients (i.e., using non-saturated fats instead of saturated fats, less salt, etc.). However, nowadays this behavior is difficult to follow because consumers are searching for convenience and consumption of convenient food products is steady increasing [7]. In this context, another way in which consumers can improve the quality of their diet while satisfying their need for convenience is to choose processed food with claims of improved nutritional and health characteristics, for example ‘fat free’. The presence of food products with nutritional and/or health claims in the market has risen because food companies are increasingly offering healthier processed food alternatives to fulfill consumers’ desires to follow a healthier diet. From the institutional point of view, the European Union has introduced certain regulations for the nutritional and health information on food products’ labels to avoid misleading consumers [8]. This regulation applies to all nutrition and health claims on processed food, indicating that nutritional and health claims (NHCs) should not be misleading and should be substantiated by generally accepted scientific data.

Several papers have analyzed the prevalence of different nutritional and health claims in different European markets [9,10,11,12]. Except for the study by Hieke et al. (2016) [9], which includes several European countries (Germany, Slovenia, Spain, the Netherlands, and the United Kingdom), the papers focus only on Ireland, the UK, and Slovenia, respectively. Hieke et al. (2016) [9] found that 26% of foods carried at least one nutritional (21%) or health claim (11%). In addition, 64% of the claims were nutritional, with the highest proportion corresponding to Spain (74%). Foods carrying claims tended to carry multiple claims, with an average number of nutritional claims per product of 2 in the European countries and 2.1 in Spain. Apart from vitamins and minerals, the nutritional claims mainly referred to fat content (24%), followed by sugar (12%) and fiber (9%).

Thus, consumers have access to healthier food product alternatives that could help them to follow a healthier diet. However, making information available and ensuring that this information is correct is only the first step in inducing healthier diets. For consumers to choose healthier food products, it is necessary that they value positively the nutritional and/or health claims and are willing to buy and pay for them. Several empirical papers analyzed consumers’ acceptance of food products with health-related claims by studying motivations, attitudes, and intention to purchase food products with these claims [13,14,15]. However, only a few papers were conducted to investigate consumers’ preferences for them and to assess consumers’ valuation for nutritional claims [16,17,18,19]. The latter papers estimated the extra price consumers are willing to pay for different nutritional and health claims for different products. All of them found that consumers would pay an extra price for a food product with nutritional or health claims. Accordingly, the objective of this paper is to investigate consumers’ preferences for two relevant nutritional claims in Spain: in other words, to assess consumers’ valuation for these claims. In addition, we are mainly interested in the possible differences in consumers’ valuation of nutritional claims and in explaining these differences. This will allow us to profile consumers in terms of their willingness to pay for nutritional claims. This second objective is highly relevant because the analyzed nutritional claims exist in the market and the characterization of consumers would allow food companies and public authorities to tailor their marketing and information strategies and educational programs to help consumers to buy healthier food alternatives.

Spain was selected because of the higher number of nutritional claims in its market. The selection of the food product and the nutritional claims analyzed was made simultaneously based on the previous studies on the prevalence of nutritional and health claims in the market and on direct observations of the shelves in different supermarkets. As mentioned above, the most prevalent nutritional claims in the market are those related to fat content, sugar, and fiber. Hieke et al. (2016) [9] also indicated that, apart from baby and dietary foods, the cereal food category had the highest proportion of nutritional claims (30%). Therefore, a direct observation of different supermarkets was conducted to gather information on the different nutritional claims available in different cereal products (biscuits, toast bread, and breakfast cereals). Based on this information, we chose breakfast biscuits due to the high prevalence of nutritional claims and because they are a highly consumed product in Spain. Within breakfast biscuits, the most frequent nutritional claims were related to fiber. In addition, the evidence to date indicates that high-fiber diets are beneficial for weight control and protective of the development of diabetes and heart disease [6]. Accordingly, we chose the nutritional claim ‘high in fiber’. Second, we were interested in the reduction of saturated fats (SFA) because it has been scientifically proven that reducing the intake of saturated fats and replacing them with polyunsaturated fatty acids (PUFA) reduces the risk of some health diseases related to diet, in particular cardiovascular diseases [20]. Thus, we chose the ‘reduced saturated fat’ claim as our second claim to analyze, although this claim was only used in a few breakfast biscuits. This fact also supports our decision because consequently we analyze a claim with high prevalence in the market (‘high in fiber’) and another with low prevalence (‘reduced saturated fat’).

To assess consumers’ valuation of the ‘high in fiber’ and ‘reduced saturated fat’ claims, we designed a non-hypothetical artefactual choice experiment and implemented it in a close-to-real environment (mock/real brick-and-mortar supermarket). The experiment was carried out in a Spanish town where this mock/real brick-and-mortar supermarket was located in June 2015 with a sample of consumers stratified by age, gender and body mass index.

## 2. Materials and Methods

We used an artefactual non-hypothetical experiment conducted in a realistic setting, specifically a mock/real brick-and-mortar supermarket, to increase the external validity of the study.

### 2.1. Study Design

First, we decided to use an artefactual experiment [21] to ensure that the recruited participants were representative food purchasers and had experience with the concerned good [22]. In addition, to ensure that the respondents had experience with the good, the target population consisted of participants who were responsible for the purchase of food products in their household and who consumed breakfast biscuits.

Second, we decided to conduct a choice experiment instead of other valuation methods because of its ability to value multiple attributes simultaneously, its consistency with the random utility theory, and the similarity of the choice task asked of the participants to their real purchase decisions [23]. In addition, we designed a non-hypothetical experiment instead of using a hypothetical choice experiment to avoid hypothetical bias. Several papers have analyzed the hypothetical bias in choice experiments and compared results from both hypothetical and non-hypothetical versions ([24,25,26,27], among others). They have all provided strong evidence of hypothetical bias, suggesting the use of non-hypothetical experiments. In addition, Chang et al. (2009) [24] found that non-hypothetical choices are a better approximation of true preferences than hypothetical ones, based on a comparison not only between hypothetical and non-hypothetical choice experiments but also with actual market shares. The interpretation of this finding is that the willingness to pay (WTP) values of non-hypothetical choice experiments (CEs) can be assumed to be the true values corresponding to actual payments in the marketplace [24].

**Figure A1:**
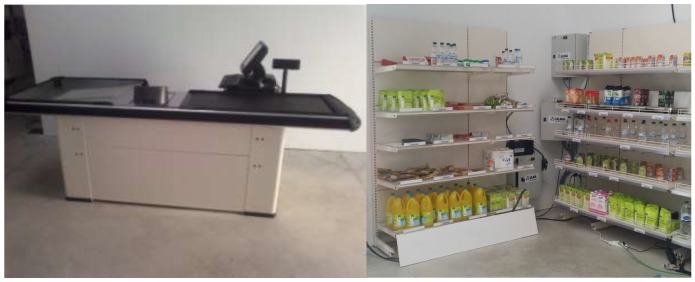
Smart Point of Sale for experimental settings.

Third, as the proposed research has an empirical orientation to provide stakeholders (private and public) with information on consumers’ valuation of different nutritional claims, the external validity of the experiment is vital to allow for the generalization of the results. To increase the external validity of our experiment by increasing its ecological validity, the choice experiment was carried out in a close-to-real setting. In other words, we used a setting as similar to a real supermarket as possible. In particular, we used a mock/real brick-and-mortar supermarket. This mock/real supermarket is located in a logistic facility in the town where the experiment took place. This logistic space is available for companies to demonstrate how their product and service technology can help to create innovative solutions and improve productivity and competitiveness in the field of logistics. The logistic demonstration center is divided into several modules; Smart Store, Smart Point of Sale, Supply Chain Module, and Intelligent Transport Module. In this research, we used the Smart Point of Sale, which consists of a Smart Point of Sale Terminal and Smart Shelving for automatic inventory control to undertake the experiments in a close-to-real environment (Figure A1 in the Appendix A).

We designed a non-hypothetical choice experiment, introducing real economic incentives and real products. The participants received €10 because at the end of the experiment one choice set was randomly selected as binding and the respondents were required to purchase the food product chosen in the binding situation at the corresponding price. 

We selected a box of half a kilo of breakfast biscuits with three different attributes: price, fiber, and fat content claims. The price levels were set, based on the market prices at the time of the experiment, at 0.5 €/box, 1.5 €/box, 2.5 €/box, and 3.5 €/box. The other two attributes had two options; the product carried the claim ‘high in fiber’ (FIBER) or the claim ‘reduced saturated fat’ (FAT) or did not carry a claim. The attributes selected and their levels are summarized in Table 1.

The choice set design was generated following the Street and Burgess (2007) [28] approach. For the main effects, three attributes were chosen with four, two, and two levels, respectively, as well as two options; we obtained eight pairs, and this design was 96.66% efficient compared with the optimal design. Thus, each respondent was asked to make four choices because we randomly split the choice sets into two blocks. Each choice set included three alternatives; two designed alternatives consisting of different products and a non-buy option.

### 2.2. Participants and Recruitment

The experiment was conducted in June 2015 in a medium-sized town in Spain where the mock/real supermarket is located. This town is widely used by food marketers and market research consulting companies since its socio-demographics are representative of the Spanish Census of Population (Table A1 in the Appendix A). The target population was consumers who were responsible for the purchase of food products in their households and who consumed breakfast biscuits. The participants were recruited by an external company using our requirements: (i) food shoppers; (ii) breakfast biscuit consumers; and (iii) stratified by age, gender, and body mass index. The sample size for the experiment was chosen based on similar real experiments for agro-food products [29,30,31,32,33]. A total of 121 consumers stratified by gender, age, and body mass index participated in the experiment.

### 2.3. Implementation Procedure

The experiment was conducted by the research team as follows. On arrival, the consumers received information on the nature of the experiment and signed informed consent for participation. An ID number was assigned to each respondent to guarantee anonymity. The monitor provided a general overview of the working session and informed the participants that, at the end of the experiment, they would receive €10 to purchase a box of biscuits (the one that they chose in the binding choice set) at the corresponding price. The monitor insisted that it was in their best interest to choose only the product that they were really interested in purchasing because this product could be the selected type of biscuit in the binding choice set. In addition, the participants received all this information in clear written instructions together with the information on the biscuits and the attributes presented in the different choice tasks. Then, the respondents were asked to choose four times between two boxes of biscuits or the non-buy option in front of the supermarket shelves with the real biscuit boxes (Figure A1 in the Appendix A). Afterwards, they went to the cashier and another monitor asked them to select randomly one card out of four cards numbered from 1 to 4 (choice sets) to determine the binding choice set (see Figure A1 in the Appendix A). Then, the respondent received €10 to purchase the box of breakfast biscuits selected in this binding choice set at the corresponding price.

The respondents were also required to complete a brief questionnaire with the following structure: (i) food and breakfast product purchase and consumption; (ii) objective nutritional knowledge and use of nutritional information; (iii) interest in healthy eating; (iv) weight, height, and health status; and (v) socio-demographic and economic characteristics (gender, family size and composition, age, educational level, and income range).

### 2.4. Measures

The measurement of consumers’ choice were made by asking the respondents to choose four times between two boxes of biscuits or the non-buy option in front of the supermarket shelves, as mentioned above. In the questionnaire the participants were asked first whether they were responsible for food purchases. In addition, consumers were required to rate on a seven-point scale the importance that they attached to different attributes when shopping for food products. Finally, the respondents were asked about their frequency of consumption of cereals and breakfast biscuits; the options included never/once a month or less, 2–3 times a month, 1–2 times a week, 3–4 times a week, 5–6 times a week, once a day, and more than once a day.

The objective nutritional knowledge was measured following Grunert et al.’s (2010) [34] scale based on the knowledge of dietary recommendations. The participants were asked about their knowledge of health expert recommendations (should eat more, about the same, less, or try to avoid) regarding a series of nutrients or substances. 

The use of nutritional information was assessed using four items on a seven-point Likert scale (e.g., ‘I usually pay attention to nutrition information when I see it in an ad or elsewhere’) based on Moorman (1998) [35]. The Cronbach’s alpha for both four-item measures was 0.78, indicating good internal consistency reliability. Interest in healthy eating was measured on a seven-point Likert scale using the Roininen et al. (1999) [36] scale (e.g., ‘The healthiness of food has little impact on my food choices’) The Cronbach’s alpha for both eight-item measures was 0.84, indicating very good internal consistency reliability.

Finally, the participants were required to report, apart from their socio-demographic and economic characteristics, their weight, height, and health problems. With this information, each participants’ Body Mass Index (BMI) was calculated, and the participants were classified into different groups following Aranceta-Bartrino et al. (2016) [37]. Details on how each item is measured can be found in the results section.

### 2.5. Data Analysis: Discrete Choice Modeling

The data gathered in the choice experiment were used to estimate a utility function derived from the Lancastrian consumer theory of utility maximization [38]. Lancaster (1966) [38] proposed that the total utility associated with the provision of a good can be decomposed into separate utilities for their attributes. However, this utility is known to the individual but not to the researcher. The researcher observes some attributes of the alternatives, but some components of the individual utility are unobservable and treated as stochastic (random utility theory by McFadden, 1974) [39]. Thus, the utility is taken as a random variable for which the utility from the *n*th individual facing a choice among *j* alternatives within choice set *J* on each of *t* choice occasions is represented as follows:

(1) $$U_{njt}=\beta X_{njt}+\;\varepsilon_{njt}$$

where β is a vector of parameters associated with the vector of explanatory variables *X_njt_*, and ε*_njt_* is an independent identically distributed (i.i.d.) error term over time, people, and alternatives. Traditionally, it was assumed that consumers were homogeneous in terms of taste, and conditional logit models were fitted [39]. However, numerous empirical papers using choice experiments have found that consumers’ preferences for food products are heterogeneous. In this case the specification of the model should allow the parameters to vary in the population. Two alternatives have gained popularity when addressing this issue of heterogeneity, the random parameter logit model (RPL) and the latent class logit model (LC), both of which are versions of a mixed logit model [40]. In the RPL, each individual has a unique set of preferences and estimates of the utility function. Then, heterogeneity is included by adding a vector of parameters that incorporates individual preference deviations with respect to the mean preference values; β in (1) is not constant but varies across individuals, β*_n_*. However, if the preferences are assumed not to be ‘unique’ for each individual but rather distinct for a determined number of individual classes, the LC suits the modeling of choices better. In this model, consumers are assumed to belong to different segments or classes, each of them characterized by different class-specific utility parameters. In other words, within each segment, consumers’ preferences are homogeneous, but preferences vary between segments, reflecting a ‘lumpy’ spread of preferences and allowing a more in-depth understanding of heterogeneity [40]. The latter modeling approach has gained popularity and has recently been used in several studies on consumers’ valuation of food products [41,42,43,44,45,46].

In the LC model, the utility of individual ‘*n*’ choosing alternative *j* on the *t*th choice occasion is:

(2) $$U_{njt|S}=\beta_{S}X_{njt}+\;\varepsilon_{njt|S}$$

where β*_s_* is the parameter vector of class *s* associated with the vector of the explanatory variable, and *X_njt_* and ε*_njt|s_* are error terms that follow a Type I (or Gumbel) distribution. 

Thus, the probability that an individual will select alternative *j*, conditional on being in segment *s*, can be expressed as follows:

(3) $$P_{nj}=\overset{S}{\underset{s=1}{\sum }}P_{ns}\overset{T}{\underset{t=1}{\prod }}P_{njt|s}$$

where *P_ns_* is the allocation of individual *n* to the *s* class (probability of class *s*), and *P_njt|s_* is the choice probability that individual *n*, conditional on belonging to class *s* (*s* = 1, …, *S*), chooses alternative *j* from a particular set *J*, comprising *j* alternatives, on a particular choice occasion *t* [47].

The parameters for the attributes are estimated by maximizing the likelihood function in the state of incomplete prior information on class membership or choice probabilities [46]. Then, the number of segments is endogenously determined jointly with the utility coefficients. The latent class model was estimated using NLOGIT 5.0 (Econometric Software Inc., Plainview, NY, USA). 

In our empirical specification, the utility function includes the product attributes as explanatory variables, as well as an alternative-specific constant (α) representing the non-buy option. The utility function is specified as follows:

(1) $$U_{njt/S}=\alpha +\beta_{S1}PRICE_{njt}+\beta_{S2}FIBER_{njt}+\beta_{S3}FAT_{njt}+\;\varepsilon_{njt/S}$$

The constant α represents the alternative-specific constant coded as a dummy variable that takes a value of 1 for the non-buying option and a value of 0 otherwise. It is expected that the constant α will receive a negative and significant value, indicating that consumers obtain a lower level of utility when they select the non-buying option than they do when selecting the other two alternatives (A and B). The price was defined by the price levels in the design. The other two variables (FIBER and FAT) were defined as dummies.

One of the key issues in latent class modeling is the selection of the number of segments to be considered. As Swait (1994) [48] stated, the optimal number of latent segments must be selected by looking at different multiple statistical criteria but also by assessing whether additional segments provide any further economic information, with the overall aim of attaining segment parsimony. To determine the best number of classes, we calculated four information criteria; the Akaike Information Criterion (AIC), the modified Akaike Information Criterion (AIC3), the Bayesian Information Criterion (BIC), and the ρ^2^, called the Akaike Likelihood Ratio Index [49]. The preferred model should be the one with the lowest AIC, AIC3, and BIC and the highest ${\bar{\rho }}^{2}$.

Using the estimated parameters, the marginal WTP was calculated as the negative ratio of the partial derivative of the utility function with respect to the attribute of interest, divided by the derivative of the utility function with respect to the variable price.

(2) $$WTP_{Attribute}=\;-\;\frac{\frac{\partial U_{njt}}{\partial \;Attribute}}{\frac{\partial u_{njt}}{\partial Price}}\;=\;-\;\frac{\beta_{Attibute}}{\beta_{Price}}$$

The marginal *WTP* was calculated for each of the obtained segments.

## 3. Results

The final sample was representative of the Spanish population (reference population) in terms of gender, age, and body mass index, as the results of tests of differences indicate (*p*-values of 0.64, 0.167 and 0.577, respectively, in Table 2). Slightly more than half of the respondents were female (52.0%), with an average age of 47 years. However, regarding the education level, the null hypothesis of equality was rejected (*p*-value = 0.001). In particular, we can see that people with secondary education were under-represented while people with higher education were over-represented. The higher proportion of people with university education occurs frequently in studies because more educated people are more prone to participate. The under- or over-representation of the sample is a feature common to many other surveys and empirical studies [50].

### 3.1. Statistical Results

To select the number of optimal clusters, we estimated the model using two, three, and four latent classes and calculated the different information criteria presented in Table 3. The calculated information criteria were constantly decreasing or increasing. However, the improvement from two to three segments was greater than the change from three to four (Table 3). Moreover, we observed in the model for four classes that the value of the estimated parameters started to deteriorate, giving a larger standard error, which is considered as an indication to stop looking for more classes [51]. Thus, we chose the estimations of the model with three segments.

Table 4 shows the results of the LC model for three segments and of the one-segment model for comparison.

In the one-segment model, as expected, the alternative-specific constant was negative and statistically significant; therefore, consumers attained a higher utility from choosing any alternative to the non-buy option. Moreover, as expected, the price variable (PRICE) was negative and statistically significant in accordance with the economic theory indicating that increments in the price decrease consumers’ utility. The positive and statistically different from zero value of the parameter estimate for the two nutritional claims indicated that the utility for the breakfast biscuits with each of the claims was higher than that for the biscuits without claims. However, these results are not the best representation of consumers’ behavior, as the LC model with three classes was superior in terms of statistical properties. 

The estimated parameters for the three segments corroborated that heterogeneity across segments exists because the estimated values were substantially different between them, not only in magnitude but also in sign. The only estimated parameter that was consistently negative across segments was the price, although it varies considerably in absolute values. The results show that consumers in general, according to the economic theory, gain lower utility as the price of the product decreases. However, the consumers in the first segment were the most price-sensitive, while the consumers in segment 2 were the least price-sensitive. The estimated coefficients for the two nutritional claims were still positive for segment 1 and segment 2 but negative and statistically significant for segment 3. This result indicates that the utility of consumers in segment 3 for the breakfast biscuits with each of the claims was lower than that for the biscuits without claims. The contrary was still found for the consumers in segments 1 and 2. Therefore, we can conclude that the majority of consumers (36% of segment 1 plus 38% of segment 2) gain higher utility from breakfast biscuits with nutritional claims than from those without these claims, with a small group of consumers (25.8% of segment 3) presenting a higher utility for the biscuits without claims.

### 3.2. Economic Valuation Results

In order to interpret the estimated parameters, the marginal willingness to pay (WTP) was calculated for each of the segments using equation (5). The WTP is the premium or extra-price that consumers are willing to pay for the food product with the claims in relation to the food product without the claim. For instance, the WTPs for the first segment were of 1.51 €/box for the ‘high in fiber’ claim and 1.83 €/box for the ‘reduced saturated fat’ claim. These values indicate that consumers in segment 1 were willing to pay an extra-price of €1.51 for a box of breakfast biscuits with the ‘high in fiber’ claim in relation to a box without this claim. In the same way, consumers in segment 1 were willing to pay an extra-price of €1.83 for a box of breakfast biscuits with the ‘reduce saturated fat’ claim in relation to the biscuits without the claim. Then, consumers were willing to pay more for the ‘reduced saturated fat’ claim than for the ‘high in fiber’ claim. Similar results were found for segment 2 (38% of consumers) because the consumers positively valued both nutritional claims, but the extra-price they were willing to pay for the ‘high in fiber’ claim (2.28 €/box) was higher than that for the ‘reduced saturated fat’ claim (1.83 €/box). Therefore, both segments can be named ‘nutritional claim seekers,’ but the first one can be considered ‘reduced saturated fat’ lovers and the second ‘high in fiber’ lovers. Finally, segment 3 (25.8% of consumers) differs from the previous ones because the consumers presented negative WTP for both nutritional claims. This result indicated that consumers would pay for a box of breakfast biscuits without claims more than for the box with each of the nutritional claims. Consequently, segment 3 can be named ‘nutritional claim avoiders’.

### 3.3. Explaining Differences in Consumers’ Valuation

To achieve our second objective, to explain the differences in consumers’ valuation of nutritional claims, we characterized these consumer segments using the information on participants described in Section 2.3 and presented in Table 2, Table 5 and Table 6. First, we conducted a series of bivariate analyses between the three segments and all of these participants’ characteristics to test for differences among the segments. In particular, a chi-square or analysis of variance test was used depending on the nature of the characterization variables. Second, we profiled the three segments according to the characteristics found to be statistically different.

The first segment includes more women, older people, and people with a lower income level (Table 2). In addition, the proportion of consumers who are always the person responsible for the food purchases in the household is the highest (Table 5). The importance that they attach to the price when shopping is the highest, although they also give importance to health, natural ingredients, and the calorie/sugar/fat content. In addition, the proportion of consumers who eat breakfast biscuits once a day or more is smaller. The consumers in segment 1 are the least knowledgeable about saturated fat because a smaller proportion stated that experts recommend eating less saturated fat (although 80% knew). The consumers in this segment stated that they usually pay attention to and use nutritional information and read about nutrition in magazines and books (with almost 5 points on a 7-point scale). These consumers showed a high interest in healthy eating because they value healthy aspects to a greater extent and unhealthy ones to a lesser extent (Table 6). Finally, the proportion of consumers with osteoporosis or other bone problems is the highest.

The consumers in the second segment share some characteristics with the consumers in Segment 1 but differ in others. They are similar in terms of age; the importance that they give when shopping to price, health, natural ingredients, and the calorie/sugar/fat content; their attention to and use of nutritional information; and their interest in healthy eating. However, the proportion of women and lower-income households is smaller and the consumers are younger. In addition, the proportion of consumers who are always the person responsible for food purchases in the household is smaller than that in segment 1. The proportion of consumers who eat breakfast biscuits once a day or more is the largest among the segments. The consumers in Segment 2 are the most knowledgeable about saturated fat because a larger proportion stated that experts recommend eating less saturated fat. Finally, the proportion of consumers with osteoporosis or other bone problems is smaller than that in segment 1.

The third segment differs more from the previous ones. It consists of the smallest proportion of women, the youngest consumers, and the largest proportion of consumers with university studies. The proportion of consumers who are always the person responsible for the food purchases in the household is the smallest. They give the least importance when shopping to health, natural ingredients, and the calorie/sugar/fat content. In addition, the proportion of consumers who eat breakfast biscuits once a day or more is the smallest. Their knowledge about saturated fat is between the other two segments because the proportion of consumers who stated that experts recommend eating less saturated fat is average. The consumers in this segment stated to a lesser extent that they usually pay attention to and use nutritional information and read about nutrition in magazines and books than the consumers in the other two segments. These consumers showed the least interest in healthy eating because they value the healthy aspects the least and the unhealthy ones the most. Finally, the proportion of consumers without any health problems is the largest among the segments.

## 4. Discussion

This study aimed to investigate consumer preferences and WTP for two nutritional claims with different degrees of prevalence in the market and related to two different nutrients (one beneficial and the other harmful to health). Specifically, a highly prevalent claim for a beneficial nutrient (‘high in fiber’) and a less prevalent one for a harmful nutrient (‘reduced saturated fat’) were selected. The selected food carrier for the claims was breakfast biscuits. The results indicated that the general consumer positively values both nutritional claims with similar WTP. This finding is consistent with the previous results obtained by Øvrum et al. (2012) [17] and Van Wezemael et al. (2014) [18], who found that a low saturated fat claim is positively valued for cheese and beef, respectively. However, heterogeneous preferences across consumers were found, and, using a latent class model, three segments of consumers based on the WTP for the two nutritional claims were detected. Two of the segments were considered ‘nutritional claim seekers,’ because their WTP for both nutritional claims was positive. The difference between these two segments is that the consumers in the first one (36% of consumers) present a higher valuation for ‘reduced saturated fat’ than for ‘high in fiber.’ This finding is similar to those obtained by Øvrum et al. (2012) [17] and Van Wezemael et al. (2014) [18], who found that the WTP for the low saturated fat claim was higher than the WPT for other nutritional claims (low fat in the first study and iron and protein claims in the second study). By contrast, the second segment (38% of the consumers) values the ‘high in fiber’ claim more than the ‘reduced saturated fat’ claim, although the WTP for ‘reduced saturated fat’ has almost the same magnitude. Thus, the second segment is named ‘fiber lovers’ and the first ‘reduced saturated fat lovers’. On the other hand, the members of the third segment (25.8% of consumers) negatively value both nutritional claims and are considered to be ‘nutritional claim avoiders’. This last segment is characterized by being younger males with university studies who are not responsible for the food purchases in the household. These consumers give the least importance to health, natural ingredients, and the calorie/sugar/fat content when shopping. They pay less attention to nutritional information, and they stated that they use this information to a lesser extent. These consumers showed the least interest in healthy eating, and they reported that they do not have health problems related to their diet. From these results we can derive some practical implications for food companies and public authorities. As most consumers positively value both nutritional claims (74%), we propose to food companies to offer breakfast biscuits with both claims to reach the market segment willing to buy and pay for these nutritional claims. As there is still a segment of consumers with a low level of interest in healthy eating, who are not willing to pay a positive premium for nutritional claims, we can make some suggestions to public authorities to change the preferences of this unwilling group. Our recommendation is to implement educational activities promoting the importance of following a healthy diet to people’s health. These activities should focus on male and younger consumers, advising them that, although they do not yet have health problems related to their diet, healthy eating is the best way of preventing them from arising. This could induce changes in the preferences for the nutritional claims, reversing their negative valuation for them.

Another interesting result is that differences were not found among obese, overweight, and normal weight people but among consumers with different interests in healthy eating and with a different prevalence of health problems. This result indicates that the consumer’s weight status itself has no effect on the willingness to pay for nutritional claims, while health problems and an interest in following a healthy diet do.

In summary, we found that most of the consumers are willing to pay for the two nutritional claims ‘high in fiber’ and ‘reduced saturated fat’, while the rest of the consumers (a quarter) value them negatively. This result is promising for both public and private stakeholders. For public authorities, it means that the use of nutritional claims can lead to healthier diets, which will reduce the prevalence of diet-related health problems for a big part of the population. For food companies, it provides information that they can use to tailor their marketing and advertising campaigns to reach the different segments.

Finally, this work poses some limitations that could undermine the generalization of the results. In particular, the study was only conducted in one European country (Spain) with a small sample. Therefore, to increase the external validity of our experiment, similar studies should be undertaken in other European countries. Moreover, the study of preferences for the two nutritional claims was applied only to one food product, breakfast biscuits. As preferences for nutritional claims may be product-specific, the study should be replicated for other food products. 

## Figures and Tables

**Table 1:** Breakfast biscuits attributes and levels.

Attributes	Levels
Price (Euro/liter)	0.5 €/box, 1.5 €/box, 2.5 €/box and 3.5 €/box
Fiber claim	None ‘High in Fiber’
Fat claim	None ‘Reduced Saturated Fat’

**Table 2:** Sociodemographic and economics characteristics and Body Mass Index.

	Sample	Population	Segment 1	Segment 2	Segment 3
Female (%) * (0.22 (0.641)) ^1^	52.07	50.90 ^2^	63.04	48.94	39.29
Age (average) **	46.98	42.68 ^2^	50.00 ^a^	48.11 ^a^	40.14 ^b^
Age (%) (21.31 (0.167)) ^1^					
18–34 ***	24.79	24.12 ^2^	15.22 ^a^	17.02 ^a^	53.57 ^b^
35–44	19.01	20.62 ^2^	23.91	19.15	10.71
45–54	25.62	18.56 ^2^	21.74	31.91	21.43
55–64 **	10.74	14.32 ^2^	17.39	10.64	0.00
≥65	19.83	22.38 ^2^	21.74	21.28	14.29
Education level (%) (18.06 (0.001)) ^1^					
Primary	17.36	17.00 ^3^	15.22	17.02	21.43
Secondary	35.54	50.00 ^3^	39.13	38.30	25.00
Higher	47.11	33.00 ^3^	45.65	44.68	53.57
Income level (%) ^4^					
≤1500 €/month *	42.71	N/A	47.83	25.53	35.71
1501–2500 €/month	33.01	N/A	23.91	31.91	28.57
>2500 €/month	24.28	N/A	17.39	25.53	17.86
Body mass index (%) (2.89 (0.577)) ^1^					
Obese and over weight	52.07	60.90 ^5^	58.07	53.19	39.29
Normal weight	46.28	37.80 ^5^	39.13	44.68	60.71
Under weight	1.65	1.20 ^5^	2.17	2.13	0.00

Note: ***, **, * denotes statistical significance at 1%, 5%, and 10%, respectively. ^a,b^ Superscript letters indicate that group means are different for continuous variables using the Bonferroni Test and that the percentages are different for discrete variables using χ^2^-square Test. ^1^ The χ^2^-square (*p*-value) Test between the sample and the population; ^2^ INE—Padrón continuo (1 January 2015); ^3^ Education at a glance: Organisation for Economic Co-operation and Development (OCDE) Indicators, OCDE (2014); ^4^ The 14.88% of the participants don’t know or prefer not to say; ^5^ Aranceta-Bartrina et al., 2016 [37].

**Table 3:** Statistics to determine the optimal number of consumer segments.

Number of Segments	Number of Parameters (p)	Log Likelihood at Convergence (LL)	AIC ^a^	AIC3 ^b^	BIC ^c^	${\bar{\rho }}^{2}$ ^d^
2	8	−434.69	885.37	893.37	446.30	0.05
3	12	−404.53	833.06	845.06	421.95	0.10
4	16	−395.36	822.72	838.72	418.59	0.11

Note: Log likelihood evaluated at zero is −464.20. ^a^ AIC (Akaike Information Criterion) is calculated using −2(LL − p); ^b^ AIC3 (Bozdogan Akaike Information Criterion) is calculated using (−2LL + 3p); ^c^ BIC (Bayesian Information Criterion) is calculated using (−LL + (p/2)xln(N)); ^d^ ${\bar{\rho }}^{2}$ is calculated using [1 − AIC/2LL(0)].

**Table 4:** Parameter values for biscuits latent class choice model.

			Latent Classes					
Variables	One-Segment Model		Segment 1		Segment 2		Segment 3	
	Coef.	*t*-ratio	Coef.	*t*-ratio	Coef.	*t*-ratio	Coef.	*t*-ratio
α	−0.28 **	−1.99	1.406 ***	3.11	−2.282 ***	−3.80	−1.302 ***	−2.93
PRICE	−0.539 ***	−9.18	−1.752 ***	−4.56	−0.353 ***	−415	−0.674 ***	−3.44
FIBER	0.647 ***	4.91	2.647 ***	3.52	0.807 ***	3.48	−1.294 ***	−2.74
FAT	0.632 ***	4.71	3.206 ***	5.04	0.647 ***	2.60	−1.440 ***	−2.79
Segment Size ^a^			36.0 ***	6.40	38.2 ***	6.67	25.8 ***	5.30
WTPs			Coef.	*t*-ratio	Coef.	*t*-ratio	Coef.	*t*-ratio
FIBER	1.20		1.510 ***	5.24	2.285 ***	3.64	−1.921 ***	−2.03
FAT	1.17		1.829 ***	5.16	1.832 ***	3.16	−2.138 ***	−2.15

Note: ***, ** denotes statistical significance at 1% and 5%, respectively. ^a^ Estimated latent class probabilities (%).

**Table 5:** Food purchase, knowledge and use of nutritional information.

	Sample	Segment 1	Segment 2	Segment 3
Food purchase				
Who is doing the groceries in your household? Always me (%) *	37.19	47.83	36.17	21.43
Importance attached when buying food to these aspects (average)				
Convenience	4.64	4.72	4.80	4.25
Price **	5.36	5.67 ^a^	5.11 ^b^	5.29 ^b^
Health **	5.63	5.74 ^a^	5.81 ^a^	5.14 ^b^
Taste	5.79	5.85	5.74	5.75
Familiarity	4.83	4.72	5.02	4.71
Natural ingredients ***	5.29	5.37 ^a^	5.62 ^a^	4.61 ^b^
Calorie/sugar/fat content ***	5.20	5.46 ^a^	5.66 ^a^	4.00 ^b^
Consumption Frequency (once a day or more) (%)				
Breakfast cereals	17.36	23.92	17.03	10.71
Breakfast biscuits *	29.76	26.09 ^a^	44.68 ^b^	25.00 ^a^
Objective nutritional knowledge (% right answers)				
Calcium	86.26	82.61	91.49	96.43
Salt	95.87	97.83	91.49	100.0
Fiber	95.87	95.65	97.87	92.86
Saturated fats *	87.60	80.43 ^a^	95.74 ^b^	85.71 ^c^
Sugar	72.73	73.91	74.47	67.86
Calories	61.98	60.87	63.83	60.71
Fats	66.94	67.39	59.57	78.57
Use of nutritional information (%)				
I usually pay attention to nutrition information when I see it in an ad or elsewhere ***	4.88	4.96 ^a^	5.51 ^b^	3.68 ^c^
I use nutrition information on the label when making most of my food selections **	4.85	4.96 ^a^	5.19 ^a^	4.11 ^b^
I do not spend much time in the supermarket reading nutrition information *	3.62	3.28 ^a^	3.57 ^a^	4.25 ^b^
I read about nutrition in magazines and books ***	4.03	4.43 ^a^	4.43 ^a^	2.71 ^b^

Note: ***, **, * denotes statistical significance at 1%, 5%, and 10%, respectively. ^a–c^ Superscript letters indicate that group means are different for continuous variables using Bonferroni Test and that the percentages are different for discrete variables using χ^2^-square Test.

**Table 6:** Interest in healthy eating and self-reported health problems.

	Sample	Segment 1	Segment 2	Segment 3
Interest in healthy eating (average)				
The healthiness of food has little impact on my food choices	2.86	2.85	2.66	3.21
I am very particular about the healthiness of food I eat ***	4.86	5.13 ^a^	5.21 ^a^	3.82 ^b^
I eat what I like and I do not worry much about the healthiness of food	3.21	3.15	2.98	3.71
It is important for me that my diet is low in fat **	4.74	4.96 ^a^	5.00 ^a^	3.96 ^b^
I always follow a healthy and balanced diet ***	4.70	4.74 ^a^	5.13 ^a^	3.93 ^b^
It is important for me that my daily diet contains a lot of vitamins and minerals **	5.02	5.24 ^a^	5.15 ^a^	4.43 ^b^
The healthiness of snacks makes no difference to me ***	2.82	2.61 ^a^	2.45 ^a^	3.79 ^b^
I do not avoid foods, even if they may raise my cholesterol *	2.92	2.76 ^a^	2.68 ^a^	3.57 ^b^
Self-reported health problems (%)				
Overweight or obesity	28.93	30.43	29.79	25.00
Cardiovascular diseases (heart, …)	1.65	2.17	2.13	0.00
Hypertension (high blood pressure)	8.26	6.52	10.64	7.14
High levels of blood cholesterol	11.57	10.87	14.89	7.14
Diabetes	0.83	2.17	0.00	0.00
Osteoporosis or other bone problems *	15.70	23.91	12.77	7.14
None of the above *	58.68	56.52	51.06	75.00

Note: ***, **, * denotes statistical significance at 1%, 5%, and 10%, respectively. ^a,b^ Superscript letters indicate that group means are different for continuous variables using Bonferroni Test and that the percentage are different for discrete variables using χ^2^-square Test.

## Appendix A

**Table A1:** Population by sex and age in Spain and in the town (%).

		Sex		Age				
	Total	Female	Male	18–34	35–44	45–54	55–64	More than 64
Spain	46,624,382	50.90	49.10	24.12	20.62	18.56	14.32	22.38
Town	956,006	50.90	49.10	22.34	20.13	18.29	14.68	24.56

Source: Spanish Census of Population, 2015. Instituto Nacional de Estadística (www.ine.es), Spain.
